# Comparison of the Effectiveness and Safety of Metoprolol and Diltiazem in Atrial Fibrillation With Rapid Ventricular Rate Patients: A Systematic Review and Meta-Analysis

**DOI:** 10.7759/cureus.56560

**Published:** 2024-03-20

**Authors:** Abshiro H Mayow, Tanya Sinha, Mansoor Ahmad, Ye Kyaw Myint, Samyuktha Balaji, Sandipkumar S Chaudhari, Divine Besong Arrey Agbor, Areeba Khan

**Affiliations:** 1 Medicine, St. George's University School of Medicine, St. George's, GRD; 2 Medical Education, Tribhuvan University, Kirtipur, NPL; 3 Medicine, Rehman Medical Institute, Peshawar, PAK; 4 Internal Medicine, University of Medicine 1, Yangon, MMR; 5 Internal Medicine, Sri Siddhartha Medical College, Tumkur, IND; 6 Cardiothoracic Surgery, University of Alabama at Birmingham, Birmingham, USA; 7 Family Medicine, University of North Dakota School of Medicine and Health Sciences, Fargo, USA; 8 Internal Medicine, Richmond University Medical Center, Staten Island, USA; 9 Critical Care Medicine, United Medical and Dental College, Karachi, PAK

**Keywords:** meta-analysis, diltiazem, metoprolol, rate control, atrial fibrillation

## Abstract

This study aims to assess the association between intravenous diltiazem and metoprolol in rate control for atrial fibrillation (AF) patients with rapid ventricular rate, focusing on rate control efficacy and hemodynamic adverse events. Following the Preferred Reporting Items for Systematic Reviews and Meta-Analyses (PRISMA) guidelines, electronic searches were conducted in Embase, PubMed, and Cochrane Central Register of Controlled Trials (CENTRAL) until February 20, 2024. The primary outcome was achieving ventricular rate control < 110/min. Secondary outcomes included new hypotension (systolic blood pressure < 90 mm Hg) and bradycardia (heart rate < 60/min). Nineteen studies (three randomized controlled trials and 16 observational studies) were included in this meta-analysis. Pooled analysis showed intravenous metoprolol resulted in a 39% lower rate control attainment compared to diltiazem (OR: 0.61; 95% CI: 0.44 to 0.84; p = 0.002). There were no significant differences in bradycardia (OR: 0.51; 95% CI: 0.22 to 1.22; p = 0.13) or hypotension risk (OR: 1.08; 95% CI: 0.72 to 1.61; p = 0.72) between the two groups. Intravenous diltiazem demonstrated superior rate control efficacy compared to metoprolol in AF patients with rapid ventricular rate. However, no significant differences were observed in safety outcomes, namely, bradycardia and hypotension.

## Introduction and background

Atrial fibrillation (AF) is prevalent among critically ill patients in the intensive care unit (ICU) and has been linked to heightened mortality rates [1]. Notably, in a large-scale epidemiological investigation, AF was detected in 25.5% of 60,209 hospitalizations for sepsis [2]. Moreover, AF results in about 600,000 visits to the emergency department (ED), 450,000 hospitalizations, and 22,000 deaths every year in the United States [3]. Individuals diagnosed with AF encounter a notably increased likelihood of experiencing cardioembolic stroke by a factor of 5, a three-fold higher risk of heart failure (HF), and twice the mortality rate [4].

Current guidelines for managing AF advocate for intravenous administration of either a beta blocker or a non-dihydropyridine calcium channel blocker to achieve rate control in acute settings [5]. Pharmacokinetic data indicate that intravenous diltiazem has a rapid onset of action within three minutes, albeit with a duration of effect ranging from one to three hours [6]. In comparison, metoprolol administered in doses of 5 mg and 15 mg over 10 minutes demonstrates an onset of action after 20 minutes, with durations of action lasting between five and eight hours [7]. Although pharmacokinetic profiles suggest diltiazem may act more swiftly but with a shorter duration compared to metoprolol, guidelines remain equivocal regarding their preference in unstable AF.

Given the emergence of new studies since the last meta-analysis comparing diltiazem and metoprolol in AF patients, an updated meta-analysis is warranted [8]. The purpose of this review is to offer emergency physicians evidence-based guidance on choosing the suitable medication for controlling heart rate in AF patients who arrive at the emergency department with a rapid ventricular rate. The main goal is to investigate whether there is a correlation between using intravenous diltiazem or metoprolol for rate control in AF patients with rapid ventricular rate, particularly focusing on achieving effective rate control and potential adverse hemodynamic events such as hypotension and bradycardia.

## Review

Methodology

We followed the Preferred Reporting Items for Systematic Reviews and Meta-Analyses (PRISMA) guidelines during the execution of this systematic review and meta-analysis.

Search Strategy

We conducted electronic searches in databases including EMBASE, PubMed, and the Cochrane Central Register of Controlled Trials (CENTRAL) from the inception of databases to February 20, 2024. Our search utilized key terms such as "diltiazem," "metoprolol," and "atrial fibrillation," along with Boolean algebra operators and medical subject heading (MeSH) terms. Additionally, we identified supplementary studies by scrutinizing the reference lists of eligible publications. Language restrictions were not applied. Two investigators conducted the literature search, with any discrepancies resolved through discussion.

Inclusion and Exclusion Criteria

Articles were considered for inclusion if they compared the efficacy and safety of intravenous diltiazem versus metoprolol in adult patients (aged 18 years and above) with AF. Inclusion criteria mandated the utilization of clearly defined clinical outcomes pertaining to efficacy (achievement of rate control target) and safety (occurrence of hypotension or bradycardia as adverse events) associated with both medications. We encompassed both randomized controlled trials (RCTs) and non-randomized controlled trials (non-RCTs). Our primary outcome measure was the attainment of ventricular rate control <110/min in hospitalized patients with AF and rapid ventricular rate, while secondary outcomes included the incidence of new hypotension (systolic blood pressure < 90 mm Hg) and bradycardia (heart rate < 60/min) post-administration of intravenous diltiazem or metoprolol. Articles investigating oral medications for chronic rate control in outpatients with AF or focusing on the management of AF in patients with pre-excitation syndromes were excluded. Two independent reviewers screened abstracts obtained from the aforementioned search strategy based on inclusion and exclusion criteria, followed by a detailed evaluation of full-text screening. Ultimately, studies comparing metoprolol to diltiazem for AF management were included.

Data Extraction and Quality Assessment

Data extraction utilized a pre-designed data extraction sheet. Information extracted from included studies encompassed author names, publication year, study design, sample size, and outcomes assessed in this meta-analysis. One investigator entered the data into the Review Manager program (RevMan, version 5.4.1, The Cochrane Collaboration, London, UK), while another verified its accuracy. Quality assessment was performed using the Newcastle-Ottawa Scale for observational studies and the Cochrane risk of bias assessment tool for RCTs.

Data Analysis

We conducted meta-analyses using Review Manager. Dichotomous outcomes were analyzed for odds ratios (ORs) along with 95% confidence intervals (CIs) using the Mantel-Haenszel (MH) random-effects model to address clinical and statistical differences across studies. A p-value < 0.05 indicated statistical significance. Heterogeneity was evaluated visually via forest plots and through statistical tests measuring heterogeneity variance (t2) and inconsistency (I2). Significant heterogeneity was identified if I2 exceeded 50%. We performed subgroup analysis by comparing the effectiveness of two drugs to achieve the rate control in patients with heart failure with reduced ejection fraction (HFrEF).

Results

**Figure 1:**
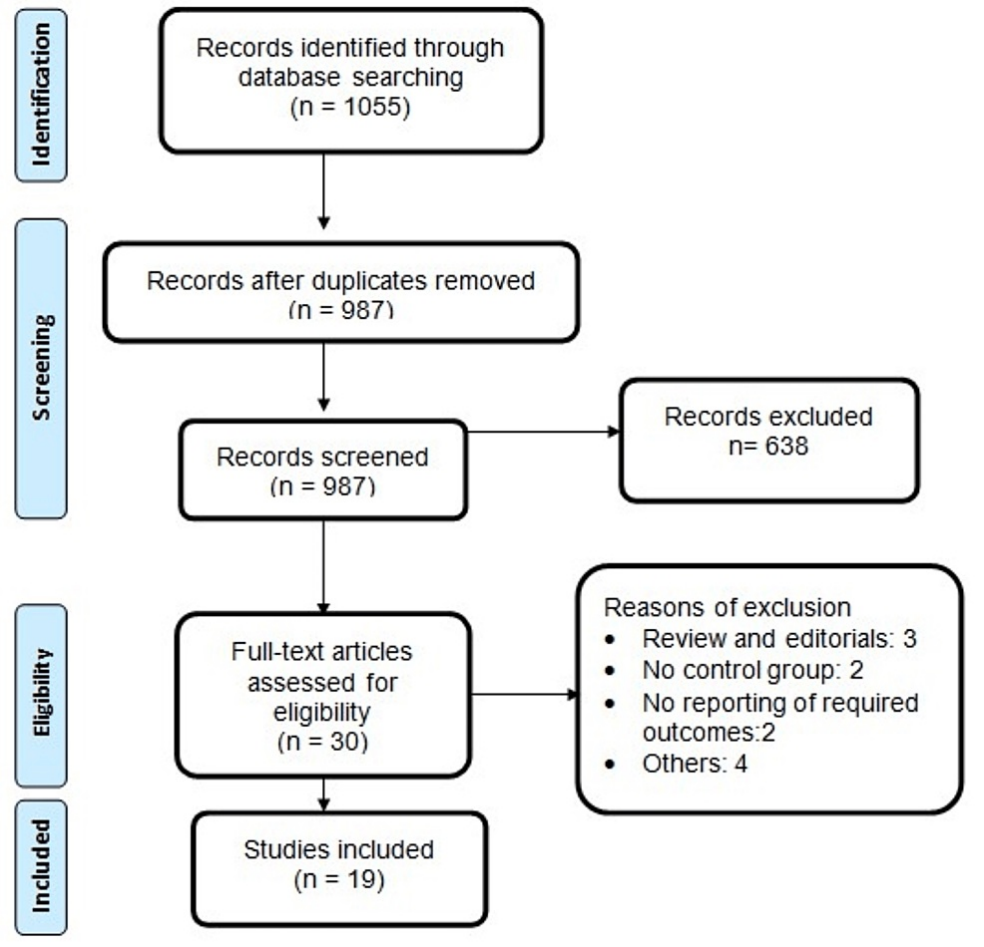
PRISMA flowchart of study selection PRISMA: Preferred Reporting Items for Systematic Reviews and Meta-Analyses.

The initial database search retrieved 1055 articles. Following the removal of 68 duplicate records, 987 unique articles underwent initial screening. Abstracts and titles were screened initially, followed by a full-text evaluation of eligible studies based on predefined inclusion and exclusion criteria. Ultimately, 19 studies met the criteria and were included in this meta-analysis, comprising a total of 2596 patients with AF and rapid ventricular rate. The study selection process is depicted in Figure 1. Of the included studies, three were RCTs, while 16 were observational in nature. The majority of studies were conducted in the United States (n = 16). Detailed characteristics of the included studies are presented in Table 1. Table 2 presents the quality assessment of the included studies.

**Table 1:** Characteristics of included studies RCT: randomized-control trial; US: United States; ED: emergency department; ICU: intensive care unit.

Author	Year	Study design	Region	Study setting	Groups	Sample size	Age	Males (n)
Blackburn et al. [9]	2023	Observational	US	ED	Metoprolol	99	67.2	57
					Diltiazem	206	65	123
Compagner et al. [10]	2022	Observational	US	ED	Metoprolol	59	75.4	20
					Diltiazem	134	72.3	52
Demir et al. [11]	2021	Observational	Turkey	ED	Metoprolol	15	NS	NS
					Diltiazem	26		
Demircan et al. [12]	2005	RCT	Turkey	ED	Metoprolol	20	NS	NS
					Diltiazem	20		
Feeney et al. [13]	2017	Observational	US	ED	Metoprolol	316	65	200
					Diltiazem	16	62	10
Fromm et al. [14]	2015	RCT	US	ED	Metoprolol	28	69.5	15
					Diltiazem	24	66.2	11
Hasbrouck et al. [15]	2022	Observational	US	ED	Metoprolol	68	65	51
					Diltiazem	57	59	44
Hargrove et al. [16]	2021	Observational	US	ED	Metoprolol	19	62.9	9
					Diltiazem	32	62.2	21
Hines et al. [17]	2016	Observational	US	ED	Metoprolol	36	65.7	24
					Diltiazem	51	64.2	27
Hirschy et al. [18]	2019	Observational	US	ED	Metoprolol	14	69	9
					Diltiazem	34	67	22
Kapustova et al. [19]	2023	Observational	US	ED	Metoprolol	15	59.8	11
					Diltiazem	30	59.2	21
Katchi et al. [20]	2014	Observational	US	ED	Metoprolol	83	NS	NS
					Diltiazem	90		
McGrath et al. [21]	2020	Observational	US	ED	Metoprolol	166	68.4	70
					Diltiazem	183	68.2	83
Medeiros et al. [22]	2021	Observational	US	ED	Metoprolol	15	76	8
					Diltiazem	51	71	22
Memis et al. [23]	2019	RCT	Turkey	ED	Metoprolol	50	73.36	NS
					Diltiazem	50	74.18	
Nicholson et al. [24]	2020	Observational	US	ED	Metoprolol	45	64	23
					Diltiazem	63	68	32
Nunez Cruz et al. [25]	2020	Observational	US	ED	Metoprolol	80	68	35
					Diltiazem	80	66	42
Personett et al. [26]	2014	Observational	US	ICU	Metoprolol	66	77	34
					Diltiazem	55	76	30
Xiao et al. [27]	2022	Observational	US	ED	Metoprolol	100	64	62
					Diltiazem	100	66	51

**Table 2:** Quality assessment of included studies

Quality assessment of observational studies						
Author	Selection	Comparison	Outcome	Overall		
Blackburn et al. [9]	3	1	3	Good		
Compagner et al. [10]	4	1	2	Good		
Demir et al. [11]	3	1	3	Good		
Feeney et al. [13]	3	1	2	Fair		
Hasbrouck et al. [15]	4	1	2	Good		
Hargrove et al. [16]	3	1	2	Fair		
Hines et al. [17]	3	1	2	Fair		
Hirschy et al. [18]	4	1	1	Fair		
Kapustova et al. [19]	4	1	2	Good		
Katchi et al. [20]	4	1	2	Good		
McGrath et al. [21]	4	1	2	Good		
Medeiros et al. [22]	4	2	1	Good		
Nicholson et al. [24]	4	1	3	Good		
Personett et al. [26]	3	1	2	Fair		
Xiao et al. [27]	4	1	2	Good		
Quality assessment of randomized control trials						
Author ID	Selection bias	Performance bias	Detection bias	Attrition bias	Reporting bias	Other bias
Demircan et al. [12]	No	No	No	No	No	No
Fromm et al. [14]	No	No	No	No	No	No
Memis et al. [23]	No	Yes	Yes	No	No	No

Meta-Analysis of Outcomes

**Figure 2:**
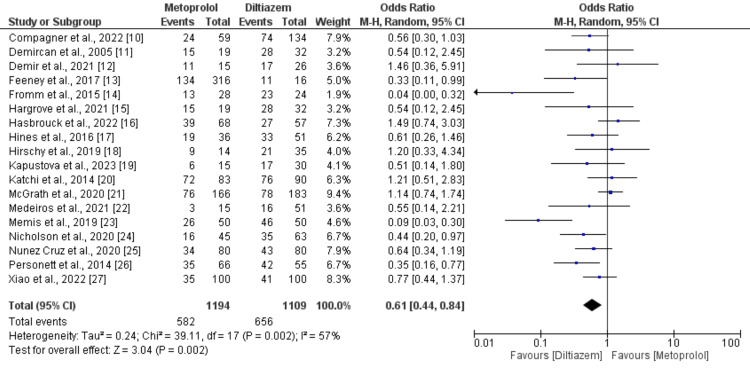
Comparison of achievement of rate control target Sources: References [10-27].

**Figure 3:**
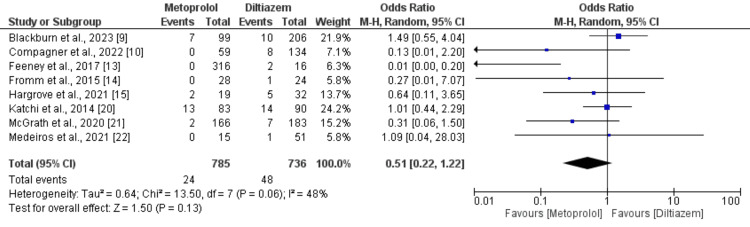
Comparison of risk of bradycardia Sources: References [9,10,13-15,20-22].

Eighteen studies, comprising 15 observational studies and three RCTs, directly assessed the attainment of rate control targets between metoprolol and diltiazem groups. Pooling data from these 18 studies, each reporting on the accomplishment of rate control target with diltiazem (n = 1109) compared to metoprolol (n=1194) in patients with AF with rapid ventricular rate, revealed that patients who received intravenous metoprolol exhibited a significantly (39%) lower attainment of rate control target compared to those treated with intravenous diltiazem (OR: 0.61; 95% CI: 0.44 to 0.84; p-value: 0.002). Notably, there was substantial heterogeneity (I-square: 57%) as illustrated in Figure 2. Additionally, eight studies, encompassing 1521 patients, reported the incidence of bradycardia as an adverse event with diltiazem versus metoprolol. A meta-analysis of outcomes from these studies indicated no significant difference in the occurrence of bradycardia as an adverse event between metoprolol (3.05%) and diltiazem (6.52%) in patients with AF with rapid ventricular rate (OR: 0.51; 95% CI: 0.22 to 1.22; p-value: 0.13), with moderate heterogeneity (I-square: 48%) depicted in Figure 3.

**Figure 4:**
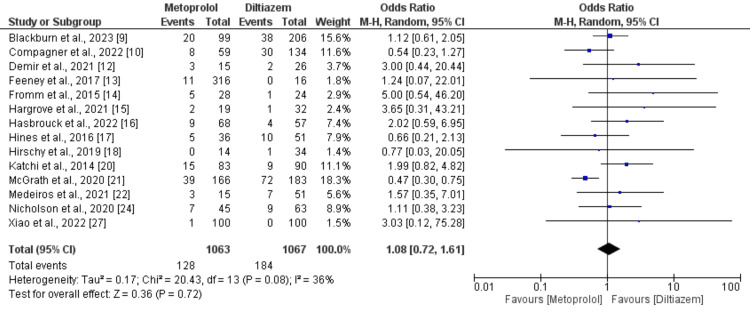
Comparison of risk of hypotension Sources: References [9,10,12-17,19-22,24,27].

**Figure 5:**
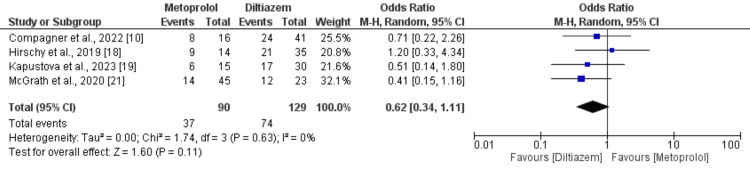
Comparison of achievement of rate control target in patients with HFrEF HFrEF: heart failure with reduced ejection fraction. Sources: References [10,18,19,21].

Moreover, 14 studies, comprising 2130 patients, documented hypotension as an adverse event associated with diltiazem versus metoprolol. Analysis of data from these studies did not uncover a significant difference in hypotension incidence between intravenous metoprolol and diltiazem in AF patients with rapid ventricular rate (OR: 1.08; 95% CI: 0.72 to 1.61; p-value: 0.72), with moderate heterogeneity (I-square: 36%), as depicted in Figure 4. Subgroup analysis was conducted, including patients with HFrEF. Only three studies encompassed patients with HFrEF, and the findings are delineated in Figure 5. The combined analysis demonstrated that patients receiving intravenous diltiazem achieved the rate control target significantly more than those treated with intravenous metoprolol. However, the results were statistically insignificant (OR: 0.62, 95% CI: 0.34 to 1.11, p-value: 0.11). No significant heterogeneity was reported among the study results (I-square: 0%).

Discussion

Our meta-analysis indicated that intravenous diltiazem proved more effective than intravenous metoprolol in managing ventricular rate in AF patients. This finding holds significance given the correlation between inadequate rate control and heightened cardiovascular adverse events [28]. However, notable heterogeneity existed among the studies. Both the “American College of Cardiology” and the “Canadian Cardiovascular Society” guidelines consider both agents as Class I recommendations for ventricular rate control in AF, without preference for any of the two drugs [29-30]. Regional variations influence the selection of agents for ventricular rate control in the emergency department, with diltiazem being predominant in North America, while metoprolol is favored in the United Kingdom and Australia [31].

Previous meta-analyses have similarly highlighted diltiazem's favorable outcomes, showing a significant increase in achieving rate control targets compared to metoprolol (OR: 1.92, 95% CI: 1.26 to 2.90) [8]. Additionally, Lan et al.'s [32] meta-analysis corroborated diltiazem's superiority, noting its higher efficacy, quicker onset time, reduced ventricular rate, and minimal impact on blood pressure. These consistent findings underscore diltiazem's suitability as the preferred medication for rate control in AF with rapid ventricular rate, providing substantial evidence for clinical decision-making.

Our study involved subgroup analysis aimed at comparing the efficacy of two drugs in patients diagnosed with HFrEF. The findings revealed that while rate control demonstrated effectiveness in patients administered diltiazem as opposed to metoprolol, no statistically significant variances were observed between the two groups. The probable rationale behind the absence of significant disparities could be attributed to the limited sample size, which consequently resulted in insufficient statistical power to discern notable differences between the groups. It is noteworthy that all four studies examining these drugs in HFrEF patients reported favorable outcomes associated with diltiazem, albeit none reaching statistical significance. This underscores the continued clinical interest in exploring the safety and efficacy profiles of diltiazem in HFrEF patients through prospective clinical trials characterized by larger sample sizes. Such endeavors hold promise for providing clearer insights into the potential benefits of diltiazem therapy in this patient population.

In our safety analysis, we meticulously assessed occurrences of bradycardia and hypotension in patients treated with diltiazem and metoprolol. Notably, our findings revealed no significant disparities between the two groups in any of the safety outcomes examined. This aligns with similar conclusions drawn from the studies conducted by Sharda et al. [8] and Lan et al. [32], wherein both meta-analyses failed to identify any substantial differences between diltiazem and metoprolol concerning incidents of bradycardia and hypotension. Consequently, our study supports the assertion that diltiazem effectively regulates heart rate without compromising patient safety. However, it is essential to note that the majority of studies included in our meta-analysis, as well as those mentioned in prior meta-analyses, were observational in nature. Hence, to affirm these findings with greater certainty, RCTs featuring larger sample sizes are imperative.

Clinical Implications

The meta-analysis underscores diltiazem's superior efficacy over metoprolol in achieving rate control for AF. Clinicians should prioritize diltiazem for acute rate control, mindful of regional practice variations. Safety profiles for both agents are comparable, emphasizing the importance of individualized therapy selection. Further research, particularly prospective trials, is warranted to confirm these findings and refine treatment guidelines, ensuring optimal management strategies for patients with AF and rapid ventricular rate.

Study Limitations

The meta-analysis predominantly comprised retrospective studies, with only three RCTs included. However, these studies were frequently constrained by factors such as selection bias, limited sample sizes, and the absence of blinding in personnel and outcome assessment. Furthermore, certain clinical outcomes, including ICU admission, mortality rates, and the use of inotropes, were not evaluated in this meta-analysis due to insufficient data within the included studies. Furthermore, there was no evaluation performed regarding the relationship between comorbidities such as coronary artery disease, hypertension, diabetes mellitus, obesity, previous stroke or transient ischemic attack, asthma or chronic obstructive pulmonary disease, infections, kidney disease, and peripheral vascular disease, and the achievement of rate control goals, as well as any adverse effects associated with the utilization of diltiazem and metoprolol. This omission was attributed to the unavailability of patient-level data for analysis. Lastly, studies were conducted either in Turkey or the United States. We need multinational RCTs to validate the findings of this study.

## Conclusions

In conclusion, our meta-analysis encompassed 18 studies, comprising 15 retrospective studies and 3 RCTs, investigating the efficacy and safety of diltiazem and metoprolol in managing ventricular rate in patients with AF. The pooled analysis demonstrated that intravenous diltiazem achieved a significantly higher rate control target compared to metoprolol, indicating its efficacy in this context. However, no significant differences were observed between the two drugs in terms of safety outcomes, namely, bradycardia and hypotension. Despite these findings, it is crucial to acknowledge the limitations inherent in the predominantly retrospective nature of the studies, as well as the absence of data on various clinical outcomes and comorbidities. Future research, particularly RCT with larger sample sizes, is warranted to validate these findings and inform clinical decision-making.

## References

[1] Walkey AJ, Wiener RS, Ghobrial JM, Curtis LH, Benjamin EJ (2011). Incident stroke and mortality associated with new-onset atrial fibrillation in patients hospitalized with severe sepsis. JAMA.

[2] Walkey AJ, Greiner MA, Heckbert SR (2013). Atrial fibrillation among Medicare beneficiaries hospitalized with sepsis: incidence and risk factors. Am Heart J.

[3] Jackson SL, Tong X, Yin X, George MG, Ritchey MD (2017). Emergency department, hospital inpatient, and mortality burden of atrial fibrillation in the United States, 2006 to 2014. Am J Cardiol.

[4] Stewart S, Hart CL, Hole DJ, McMurray JJV (2002). A population-based study of the long-term risks associated with atrial fibrillation: 20-year follow-up of the Renfrew/Paisley study. Am J Med.

[5] January CT, Wann LS, Alpert JS (2014). 2014 AHA/ACC/HRS guideline for the management of patients with atrial fibrillation: a report of the American College of Cardiology/American Heart Association Task Force on Practice Guidelines and the Heart Rhythm Society. J Am Coll Cardiol.

[6] (2024). Diltiazem hydrochloride. https://dailymed.nlm.nih.gov/dailymed/getFile.cfm?setid=5e36488b-8f2d-4dc9-b803-af1829e6fdd0&type=pdf.

[7] (2024). Novartis Pharmaceuticals. Metoprolol tartrate. East Hanover, NJ: Novartis Pharmaceuticals.

[8] Sharda SC, Bhatia MS (2022). Comparison of diltiazem and metoprolol for atrial fibrillation with rapid ventricular rate: systematic review and meta-analysis. Indian Heart J.

[9] Blackburn M, Edwards L, Woolum J, Bailey A, Dugan A, Slade E (2023). Metoprolol versus diltiazem in the emergency department for atrial fibrillation with rapid ventricular response. JEM Rep.

[10] Compagner CT, Wysocki CR, Reich EK, Zimmerman LH, Holzhausen JM (2022). Intravenous metoprolol versus diltiazem for atrial fibrillation with concomitant heart failure. Am J Emerg Med.

[11] Demi̇r MC, Doğan M, Polat E, Akpi̇nar G (2021). Intravenous diltiazem or metoprolol administration in the emergency department for acute rate control of atrial fibrillation patients with rapid ventricular response with unknown ejection fraction. Duzce Med J.

[12] Demircan C, Cikriklar HI, Engindeniz Z (2005). Comparison of the effectiveness of intravenous diltiazem and metoprolol in the management of rapid ventricular rate in atrial fibrillation. Emerg Med J.

[13] Feeney ME, Rowe SL, Mah ND, Barton CA, Ran R (2018). Achieving ventricular rate control in patients taking chronic beta-blocker therapy. Am J Emerg Med.

[14] Fromm C, Suau SJ, Cohen V, Likourezos A, Jellinek-Cohen S, Rose J, Marshall J (2015). Diltiazem vs. metoprolol in the management of atrial fibrillation or flutter with rapid ventricular rate in the emergency department. J Emerg Med.

[15] Hasbrouck M, Nguyen TT (2022). Acute management of atrial fibrillation in congestive heart failure with reduced ejection fraction in the emergency department. Am J Emerg Med.

[16] Hargrove KL, Robinson EE, Lusk KA, Hughes DW, Neff LA, Fowler AL (2021). Comparison of sustained rate control in atrial fibrillation with rapid ventricular rate: metoprolol vs. diltiazem. Am J Emerg Med.

[17] Hines MC, Reed BN, Ivaturi V, Bontempo LJ, Bond MC, Hayes BD (2016). Diltiazem versus metoprolol for rate control in atrial fibrillation with rapid ventricular response in the emergency department. Am J Health Syst Pharm.

[18] Hirschy R, Ackerbauer KA, Peksa GD, O'Donnell EP, DeMott JM (2019). Metoprolol vs. diltiazem in the acute management of atrial fibrillation in patients with heart failure with reduced ejection fraction. Am J Emerg Med.

[19] Kapustova K, Phan B, Allison-Aipa T, Choi M (2023). Acute rate control with metoprolol versus diltiazem in atrial fibrillation with heart failure with reduced ejection fraction. Am J Emerg Med.

[20] Katchi F, Nagabandi S, Shuster J, Novak E, Joseph S (2014). Treating rapid atrial fibrillation in acute decompensated heart failure: metoprolol and diltiazem are equally safe, yet metoprolol increases conversion to sinus rhythm. J Card Fail.

[21] McGrath P, Kersten B, Chilbert MR, Rusch C, Nadler M (2021). Evaluation of metoprolol versus diltiazem for rate control of atrial fibrillation in the emergency department. Am J Emerg Med.

[22] Medeiros T, Bui V, Almekdash MH, Keesari R, Lee YR (2021). Rate control with intravenous diltiazem, verapamil, and metoprolol in acute atrial fibrillation with rapid ventricular rate. SAGE Open Med.

[23] Memiş MB, Rohat AK, Öztürk TC, Özge ON, Özgür OR (2019). Which one is the first choice for rapid ventricular rate atrial fibrillation in emergency department: metoprolol or diltiazem? A randomized clinical trial. J Surg Med.

[24] Nicholson J, Czosnowski Q, Flack T, Pang PS, Billups K (2020). Hemodynamic comparison of intravenous push diltiazem versus metoprolol for atrial fibrillation rate control. Am J Emerg Med.

[25] Nuñez Cruz S, DeMott JM, Peksa GD, Slocum GW (2021). Evaluation of the blood pressure effects of diltiazem versus metoprolol in the acute treatment of atrial fibrillation with rapid ventricular rate. Am J Emerg Med.

[26] Personett HA, Smoot DL, Stollings JL, Sawyer M, Oyen LJ (2014). Intravenous metoprolol versus diltiazem for rate control in noncardiac, nonthoracic postoperative atrial fibrillation. Ann Pharmacother.

[27] Xiao SQ, Ibarra F Jr, Cruz M (2022). Intravenous metoprolol versus diltiazem for rate control in atrial fibrillation. Ann Pharmacother.

[28] Wong BM, Green MS, Stiell IG (2020). Rate control management of atrial fibrillation with rapid ventricular response in the emergency department. Can J Cardiol.

[29] Fuster V, Rydén LE, Cannom DS (2011). 2011 ACCF/AHA/HRS focused updates incorporated into the ACC/AHA/ESC 2006 guidelines for the management of patients with atrial fibrillation: a report of the American College of Cardiology Foundation/American Heart Association Task Force on practice guidelines. Circulation.

[30] Stiell IG, Macle L (2011). Canadian Cardiovascular Society atrial fibrillation guidelines 2010: management of recent-onset atrial fibrillation and flutter in the emergency department. Can J Cardiol.

[31] Rogenstein C, Kelly AM, Mason S, Schneider S, Lang E, Clement CM, Stiell IG (2012). An international view of how recent-onset atrial fibrillation is treated in the emergency department. Acad Emerg Med.

[32] Lan Q, Wu F, Han B, Ma L, Han J, Yao Y (2022). Intravenous diltiazem versus metoprolol for atrial fibrillation with rapid ventricular rate: a meta-analysis. Am J Emerg Med.

